# Vertical ridge augmentation using a porous composite of uncalcined hydroxyapatite and poly-DL-lactide enriched with types 1 and 3 collagen

**DOI:** 10.1186/s40729-019-0167-5

**Published:** 2019-05-01

**Authors:** Norio Akino, Noriko Tachikawa, Takayuki Miyahara, Reo Ikumi, Shohei Kasugai

**Affiliations:** 1 Implant Dentistry, Dental Hospital, Tokyo Medical and Dental University, 113-8510 1-5-45, Yushima, Bunkyo-ku, Tokyo, Japan; 2 Oral Implantology and Regenerative Dental Medicine, Tokyo Medical and Dental University, 113-8510 1-5-45, Yushima, Bunkyo-ku, Tokyo, Japan

**Keywords:** Collagen, Dental implants, Osteogenesis, Poly-DL-lactide, Vertical ridge augmentation, Uncalcined hydroxyapatite

## Abstract

**Background:**

Previous studies have shown that porous composite blocks containing uncalcined hydroxyapatite (u-HA; 70 wt%) with a scaffold of poly-DL-lactide (PDLLA, 30 wt%) are biodegradable, encourage appropriate bone formation, and are suitable for use as a bone substitute in vertical ridge augmentation. The present study aimed to accelerate osteogenesis in vertical ridge formation by adding types 1 and 3 collagen to the u-HA/PDLLA blocks and assessing the effect.

**Material and methods:**

The bone substitute in the present study comprised porous composite blocks of u-HA (70 wt%) with a PDLLA (27–29 wt%) scaffold and enriched with types 1 and 3 collagen (1.7 ~ 3.4 wt%). The control blocks were composed of u-HA (70 wt%) and PDLLA (30 wt%). The materials were formed into 8-mm diameter, 2-mm high discs and implanted onto the cranial bones of six rabbits. The animals were sacrificed 4 weeks after implantation, and histological and histomorphometrical analyses were performed to quantitatively evaluate newly formed bone.

**Results:**

New bone formation occurred with both block types, showing direct contact with the original bone. Mean ± standard deviation bone formation was significantly greater in the experimental blocks (25.6% ± 4.8%) than in the control blocks (17.0% ± 4.7%).

**Conclusions:**

Histological and histomorphometrical observations indicated that new bone was formed with both block types. The u-HA/PDLLA block with types 1 and 3 collagen is a more promising candidate for vertical ridge augmentation than the u-HA/PDLLA alone block.

## Background

Dental implants are widely used to restore missing teeth and have become an essential treatment modality with a high success rate [1, 2]. In fact, advances in dental implant techniques and bone substitution materials have contributed to an increase in the restoration of partially and totally edentulous patients. However, to achieve the goals of implant dentistry, hard and soft tissues of adequate volume and quality to support the implant need to be present. In particular, inadequate bone volume or quality can compromise functional and esthetic treatment outcomes [3–10], because clinicians are forced to place implants in positions where not enough bone is available. For this reason, techniques and materials that promote predictable regeneration have become necessary [11]. For instance, some patients may require an additional bone graft at the site of insufficient bone volume to ensure predictable long-term function and good esthetic treatment outcomes, and various surgical techniques that improve bone volume have therefore been presented in the literature [11, 12].

In the case of severely resorbed alveolar ridge, various augmentation operative procedures can be used to provide sufficient bone volume for reliable placement of dental implants. These techniques include extraction socket defect grafting, horizontal ridge augmentation, and vertical ridge augmentation. Regarding ridge augmentation specifically, autografts are the most predictable and successful bone grafting material [13–16]. They are more biocompatible and have a lower complication rate than other grafting materials. However, autografts have many drawbacks that may cause a patient to reject them as a donor source in clinical practice: they have limited availability and a high resorption rate, and they necessitate harvesting surgery, which is associated with morbidity, bleeding, and a risk of nerve injury [17, 18]. Alternative materials have been used to overcome these limitations, such as synthetic bone grafts, xenografts, allografts, or a combination of these [17].

Biodegradable polymers have gained widespread attention as scaffold materials in tissue engineering and have a wide range of mechanical and physical properties that can be engineered appropriately to promote tissue regeneration. For instance, porous scaffolds are capable of carrying bioactive molecules and extracellular matrix [19], and delivering signal molecule proteins via poly-DL-lactide (PDLLA) induces a prominent increase in bone volume [20, 21].

Previous studies have demonstrated the biodegradable and osteogenic properties of porous composite blocks comprising uncalcined hydroxyapatite (u-HA; 70 wt%) with a scaffold of PDLLA (30 wt%). These composite blocks are biodegradable and result in appropriate bone formation. Thus, they may be useful as a material in vertical ridge augmentation [22]. However, because PDLLA is highly hydrophobic, it is unconducive to cell invasion and proliferation [23]. Therefore, the present study aimed to accelerate osteogenesis by adding collagen types 1 and 3 to u-HA/PDLLA blocks, as well as to assess the effect of this collagen enrichment on vertical ridge augmentation.

## Methods

### Materials

**Fig. 1:**
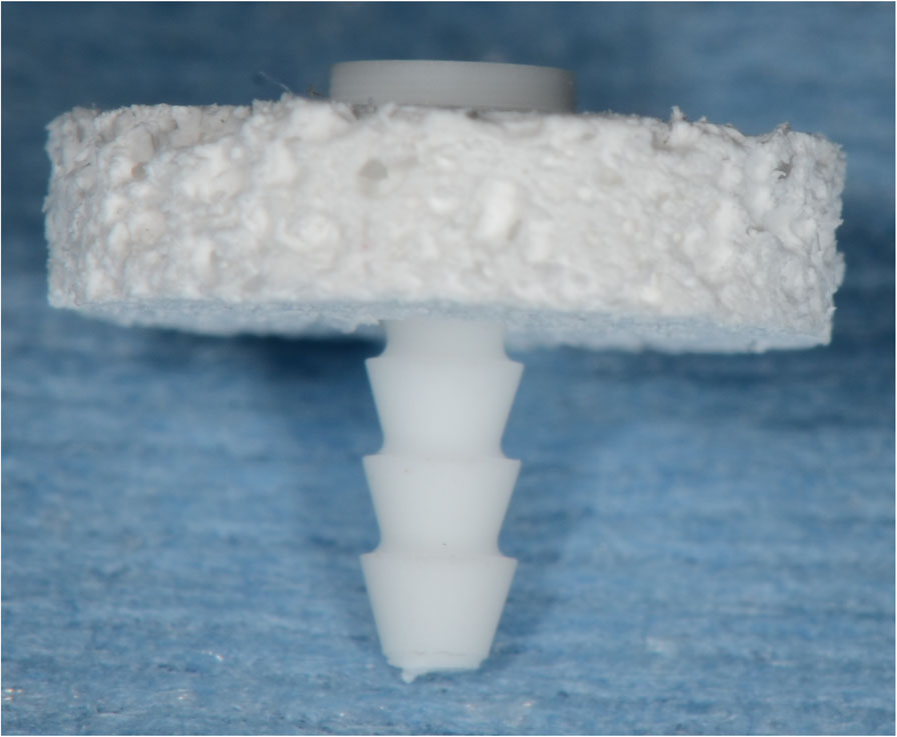
Graft materials. Fixation pin u-HA/PLLA (1.5 mm in diameter × 6 mm in length). u-HA/PDLLA composite block (8 mm in diameter× 2 mm in height)

The experimental material comprised porous composite blocks of u-HA/PDLLA enriched with types 1 and 3 collagen (porosity calculated from an apparent density of 70% and a pore diameter of 40–480 μm [average 170 μm]). The blocks of u-HA/PDLLA were dipped into types 1 and 3 collagen solution (NMP Collagen PS; NH foods Ltd., Ibaraki, Japan/concentration: 2%) with phosphate-buffered saline under reduced pressure using syringe barrel. The approach under reduced pressure was kept to dip for 20 min under ordinary temperature. After that, the blocks were dried under low temperature. The chemical composition was as follows: u-HA, 70 wt%; PDLLA (Mw 77 kDa), 27–29 wt%; types 1 and 3 collagen 1–3 wt% (NMP Collagen PS; NH foods Ltd., Ibaraki, Japan). The material was formed into discs measuring 8 mm in diameter × 2 mm in height (Fig. 1). The compressive strength of this material was close to that of cancellous bone (4.1 MPa). Control blocks were composed of a u-HA (70 wt%)/PDLLA (30 wt%) composite. Fixation pins made from composites of u-HA particles and poly-L-lactic acid (super-Fixsorb®; Takiron Co., Ltd., Osaka, Japan) were used to fix the discs (Fig. 1).

### Surgical procedure

**Fig. 2:**
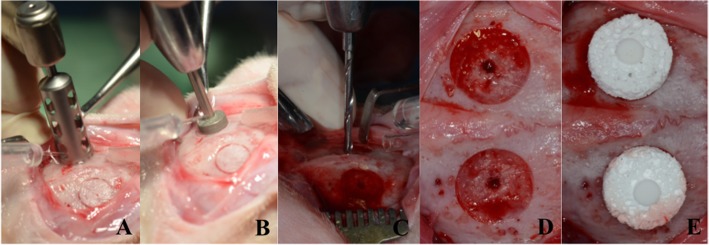
Surgical procedure. Creation of the recipient bed. The calvarial bone is marked by an 8-mm trephine bur (**a**). The cortical bone is cleared using burs (**b**). The area is rinsed with saline to remove bone debris and the center of the recipient bed is perforated with a 1.3 mm drill bit (**c**). The recipient beds are finished preparation (**d**).The blocks are positioned and fixed with fixation pins at each of the prepared sites (**e**)

Experimental protocols were approved by the Institutional Committee of Animal Care and Use at Tokyo Medical and Dental University (Approval Number: 0160314A). The experimental and control materials were implanted onto the cranial bone of six Japanese male white rabbits weighing 3.2–3.8 kg. Specifically, the surgical area was shaved and disinfected with iodine to ensure aseptic conditions. The surgery was performed under local anesthetic (2% xylocaine/epinephrine, 1:80,000; Dentsply Sankin, Tokyo, Japan). A linear incision was made from the nasal bone to the midsagittal crest. A recipient bed was then created by marking the calvarial bone using an 8-mm trephine bur and clearing the cortical bone using burs (Fig. 2a). The area was rinsed with saline to remove bone debris, and the center of the recipient site was perforated with a 1.3-mm drill. The samples were positioned and fixed using fixation pins at each of the placement areas (Fig. 2b). Thereafter, the wounds were closed with sutures. The periosteum (pericranium) and skin were then closed in layers using non-absorbable 5–0 and 4–0 nylon sutures, respectively. Animals were sacrificed 4 weeks later using a lethal dose of thiopental sodium. The entire cranial bone was removed and fixed in 10% neutral formalin for 7 days.

### Histological analysis

The sample blocks were decalcified in Plank–Rychlo’s solution for 2 days and then embedded in paraffin. The samples were sectioned parallel to the sagittal axis. Analysis under a light microscope was performed following hematoxylin and eosin (HE) staining.

### Histomorphometric analysis

Bone formation was evaluated using quantitative methods after HE staining. Each section was observed under a light microscope, and ImageJ software (National Institutes of Health, Bethesda, MD, USA) was used for image processing. Histological and histomorphometrical analyses were performed to quantitatively evaluate the percentage of newly formed bone, which was calculated using the following equation: (newly formed bone volume [%]) = (area of new bone in the material)/(implant material area) × 100.

### Statistical analysis

Statistical analysis was performed using SPSS v. 18.0 for Windows (SPSS Inc., Chicago, IL, USA). To compare the newly formed bone volume between the control and experimental groups, one-way analysis of variance was performed on the data obtained from the histomorphometric analysis. Significant differences were evaluated using the least significant difference test. The results were expressed as mean ± standard deviation (SD). Differences between the groups were assessed using the Mann–Whitney *U* test. *P* values < 0.05 were considered significant.

## Results

### Light microscopic observations

**Fig. 3:**
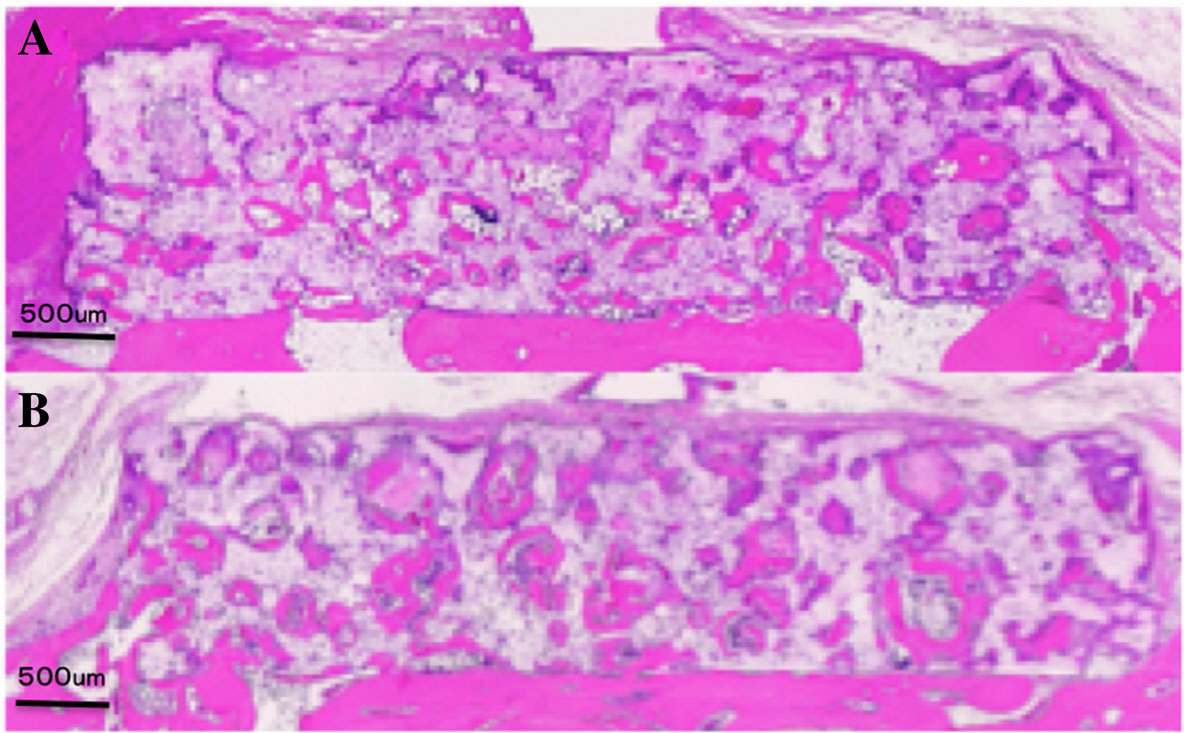
Histological. **a** u-HA/PDLLA + types 1 and 3 collagen. **b** u-HA/PDLLA

**Fig. 4:**
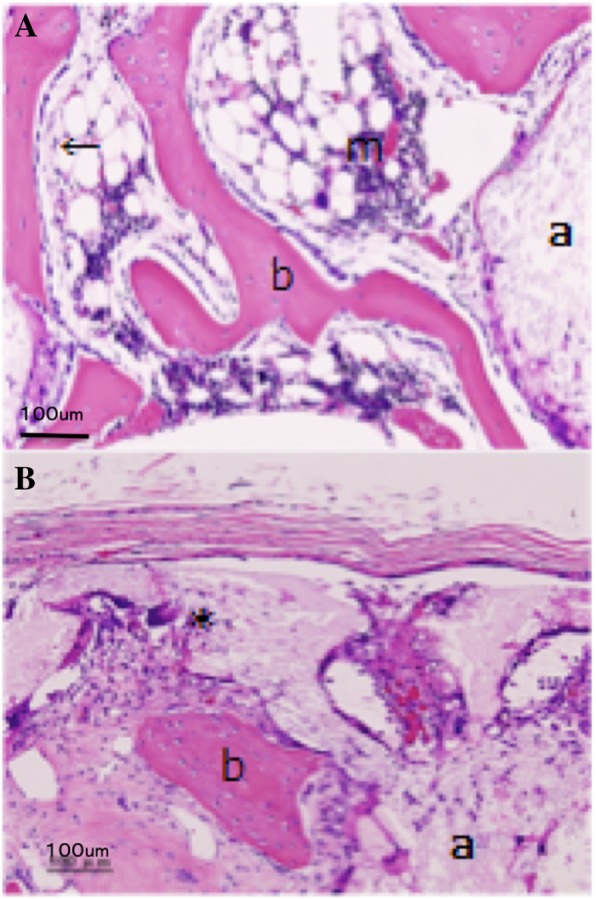
Histological (magnified image). **A** u-HA/PDLLA + types 1 and 3 collagen. **B** u-HA/PDLLA. a, material; b, newly formed bone; ←, osteoblast; m, bone marrow cell; *, foreign body giant cell

In both groups, there was direct contact between the original and newly formed bone. The bone formation had occurred in the pores, overwhelming the host bone (lower) side of both blocks. Degradation of the material was evident at the periphery. In both the control and the experimental block, the newly formed bone reached half of the material’s height (Fig. 3a, b). In a magnified image, osteoblast cells, foreign body giant cells, and the newly formed bone were observed adjacent to the block. In addition, newly formed bone and foreign body giant cell infiltration were observed in the block material, and osteoblast cells were noted adjacent to the newly formed bone (Fig. 4a, b). Although there were some inflammatory and foreign body reactions, these were infrequently observed during the period examined.

### Histomorphometry

**Fig. 5:**
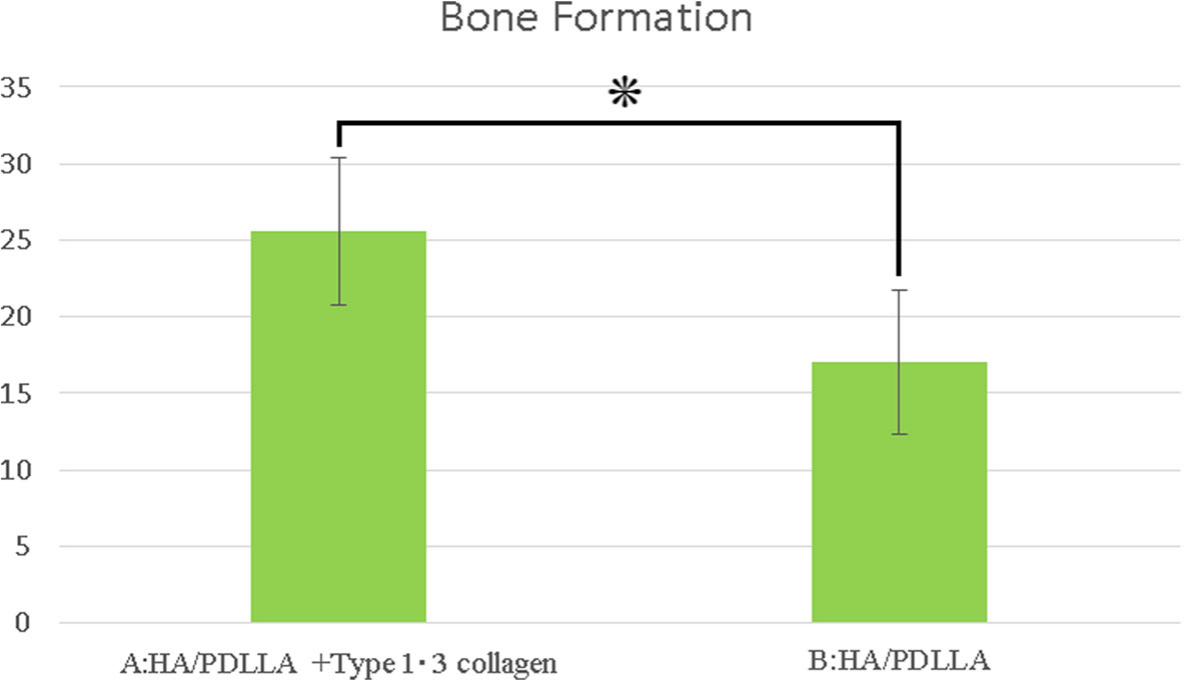
Histomorphological assessment of the newly formed bone. Bone formation (%). The date represented mean ± SD. *P < 0.05. A:HA/PDLLA + type1・3 collagen B:HA/PDLLA. There is statistically significant difference

Histomorphological assessment of the newly formed bone with both block types is shown in Fig. 5. The mean ± SD bone formation was significantly greater in the blocks composed from u-HA/PDLLA plus types 1 and 3 collagen (25.6% ± 4.8%) than in the u-HA/PDLLA control blocks (17.0% ± 4.7%) (Fig. 5).

## Discussion

The present study examined whether the hydrophilic properties of the composite material u-HA/PDLLA containing types 1 and 3 collagen increased osteogenic ability. It also assessed the efficacy of this porous material in vertical bone augmentation.

It has previously been determined that HA/PDLLA is a highly absorbent substance [22]. In the present study, the absorption appeared to occur in two phases: early and late. In the early phase, the type 1 and 3 collagen was rapidly removed, as observed in a previous experimental study involving an animal model [24]. Therefore, although large amounts of collagen were present on days 1 and 3, only traces remained at 4 weeks, and no graft remnants were found in specimens representing later intervals of healing. A previous study described the late phase of absorption, wherein the amount of u-HA/PDLLA was found to diminish significantly with time [22].

The absorption characteristics of materials depend on several parameters, including molecular architecture, the degree of crystallinity, and the copolymer ratio [19]. In the case of bioartificial materials, in vivo absorption occurs via three processes: cellular absorption by multinuclear giant cells, degradation by tissue fluids [25], and non-enzymatic hydrolysis [19], which appears to be extensive in the primary events of degradation.

In the present study, histological and histomorphometrical observations were used to evaluate the efficacy of vertical augmentation. Newly formed bone was demonstrated histologically in both the experimental and the control group. After 4 weeks, there was newly formed bone in direct contact with the surface of the block. A fibrous tissue layer was not present between the block and the host bone. The newly formed bone had infiltrated deeply into the material. Histological findings implied that the generated bone had mainly begun forming on the surface of the host bone. Virtually no newly formed bone was observed to begin forming on the periosteum side. These findings differ from those of a bone defect model, in which new bone was formed for the most part from the edge of the host bone at the base of the defect, as well as from the periosteum [13]. This difference might be due to the fact that it was difficult to achieve flap closure without tension during the external augmentation procedure. As a result, the blood supply may have been disrupted, leading to ischemia [14], which may in turn have prohibited the recruitment of osteogenic cells from the periosteum.

In the histomorphometrical assessment, the u-HA/PDLLA blocks enriched with types 1 and 3 collagen were associated with significantly greater bone formation (25.6% ± 4.8%) than u-HA/PDLLA-only blocks (17.0% ± 4.7%). Furthermore, histological and morphometric analysis indicated that collagen promoted the new formation of bone in the augmentation models. Therefore, it is likely that types 1 and 3 collagen increase osteogenic ability, and that these materials could be combined with cells and growth factors in cell therapy and tissue engineering approaches that enhance or accelerate bone repair.

Collagen constitutes the primary structural element of connective tissue, which is responsible for the functional integrity of the bone, cartilage, skin, and tendon, in which collagen accounts for most of the proteins present. It is also crucial to the structural integrity of blood vessels and most organs, where it forms a framework within which tissues can function. Importantly in the present context, collagen is present within the connective tissue of the periodontium, and a variety of conditions in humans, both healthy and pathological, involve the repair and regeneration of this collagenous framework [26]. There are two possible explanations for the significant effect of collagen addition on the bone-forming ability of the composite material. First, the surface of PDLLA is hydrophobic [23] and has low bioactivity [27]. These properties inhibit cell infiltration and osteogenesis, both of which would be accelerated by the addition of collagen. Second, collagen can be employed as a hemostatic agent and as a scaffold for bone and cartilage tissue engineering [28–30]. In fact, it is one of the most widely used bone-filling biomaterials in current bone tissue engineering [31–33]. In addition, collagen may also have bone-repairing abilities [34]. In one study, the addition of collagen to a fibrin network increased osteoblast differentiation in a dose-dependent way [35]. Furthermore, several studies have evaluated the influence of hemostatic agents on bone repair and have found both positive and negative results for collagen [34, 36].

Collagen breakdown can occur during inflammation, trauma, tissue breakdown, remodeling, and tissue repair or wound healing, and the process has two different pathways [37]: an intracellular and an extracellular route [38–40]. Under non-pathological conditions, phagocytosis, and intracellular digestion of collagen occurs continuously in dynamic soft connective tissues [41, 42]. This was corroborated in the present study by the results of surgery, which involved incision and avulsion of the periosteum and osteotomy of the cranial bone—procedures that disrupt the balance between synthesis and degradation. Indeed, even during the early phase of new bone formation, much of the collagen is broken down to make space for the infiltrating inflammatory cells, leading to swelling and redness in the tissue. It follows that sufficient collagen is necessary for tissue repair, explaining why the u-HA/PDLLA enriched with types 1 and 3 collagen resulted in a significantly more newly formed bone than the u-HA/PDLLA-alone blocks.

Collagen appears to enhance the activity of fibroblast cells themselves. When it is applied to defects of the skin or mucous membranes, vascular cells invade from the surrounding tissue. After the new structure has been formed, collagen is gradually degraded and absorbed with maturation [43]. Furthermore, collagen has a hemostatic effect that appears to be mediated through platelet agglutination and plasma component aggregation, improving tissue regeneration in wound healing and organ repair [44, 45]. It may also affect tissue healing in bone—in a perforated cortical model, collagen promoted bone augmentation, possibly by acting as a scaffold for cells and maintaining the space for bone growth [46]. Thus, it is likely that, in the present study, this tissue engineering ability promoted increased new bone formation in the experimental group.

In conclusion, u-HA/PDLLA enriched with types 1 and 3 collagen and implanted into rabbits promoted the formation of new bone. It follows that osteogenic ability is increased by the addition of types 1 and 3 collagen, and this material is a more promising candidate than u-HA/PDLLA for vertical ridge augmentation. The findings of the present study should prompt further research into this newly established approach. In particular, this material should be combined with signaling molecules that stimulate bone regeneration.

## Abbreviations

HE: Hematoxylin and eosin
PDLLA: Poly-DL-lactide
SD: Standard deviation
u-HA: Uncalcined hydroxyapatite
